# Serotype Distribution, Antimicrobial Susceptibility, and Molecular Epidemiology of *Streptococcus pneumoniae* Isolated from Children in Shanghai, China

**DOI:** 10.1371/journal.pone.0142892

**Published:** 2015-11-16

**Authors:** Fen Pan, Lizhong Han, Weichun Huang, Jin Tang, Shuzhen Xiao, Chun Wang, Huihong Qin, Hong Zhang

**Affiliations:** 1 Department of Clinical Laboratory, Shanghai Children’s Hospital, Shanghai Jiaotong University, Shanghai, China; 2 Department of Clinical Microbiology, Ruijin Hospital, Shanghai Jiaotong University School of Medicine, Shanghai, China; 3 Shanghai Children’s Medical Center, Shanghai Jiaotong University School of Medicine, Shanghai, China; 4 Shanghai Sixth People's Hospital, Shanghai Jiaotong University, Shanghai, China; Faculdade de Medicina de Lisboa, PORTUGAL

## Abstract

**Objective:**

*Streptococcus pneumoniae* is a common pathogenic cause of pediatric infections. This study investigated the serotype distribution, antimicrobial susceptibility, and molecular epidemiology of pneumococci before the introduction of conjugate vaccines in Shanghai, China.

**Methods:**

A total of 284 clinical pneumococcal isolates (270, 5, 4,3, and 2 of which were isolated from sputum, bronchoalveolar lavage fluid, blood, cerebral spinal fluid, and ear secretions, respectively) from children less than 14 years of age who had not been vaccinated with a conjugate vaccine, were collected between January and December in 2013. All isolates were serotyped by multiplex polymerase chain reaction or quellung reactions and antimicrobial susceptibility testing was performed using the broth microdilution method. The molecular epidemiology of *S*.*pneumoniae* was analyzed by multilocus sequence typing (MLST).

**Results:**

Among the 284 pneumococcal isolates, 19F (33.5%), 19A (14.1%), 23F (12.0%), and 6A (8.8%) were the most common serotypes and the coverage rates of the 7-, 10-, and 13-valent pneumococcal conjugate vaccines (PCV7, PCV10, and PCV13) were 58.6%, 59.4% and 85.1%, respectively. Antimicrobial susceptibility showed that the prevalence rates of *S*.*pneumoniae* resistance to penicillin were 11.3% (32/284). Approximately 88.0% (250/284) of the isolates exhibited multi-drug resistance. MLST analysis revealed a high level of diversity, with 65 sequence types (STs) among 267 isolates. Specifically, the four predominant STs were ST271 (24.3%, 65/267), ST320 (11.2%, 30/267), ST81 (9.7%, 26/267), and ST3173 (5.2%, 14/267), which were mainly associated with serotypes 19F, 19A, 23F, and 6A, respectively.

**Conclusions:**

The prevalent serotypes among clinical isolates from children were 19F, 19A, 23F, and 6A and these isolates showed high resistance rates to β-lactams and macrolides. The Taiwan^19F^-14 clone played a predominant role in the dissemination of pneumococcal isolates in Shanghai, China. Therefore, continued and regional surveillance on pneumococcal isolates may be necessary.

## Introduction

*Streptococcus pneumoniae*, an encapsulated Gram-positive bacterium, represents a prominent pathogen associated with various illnesses, ranging from self-limiting infections to life-threatening invasive diseases. Worldwide, approximately 1 million patients die of pneumococcal infections annually, especially children less than 5 years old [1–2]. Based on differences in the capsular polysaccharide (*cps*), more than 90 serotypes of *S*. *pneumoniae* have been identified, but only a limited number of serotypes cause the majority of severe pneumococcal infections [3].

Considering the high incidence and severity of pneumococcal diseases, researchers are dedicated to exploring new and effective methods to prevent the dissemination of *S*.*pneumoniae*. Since 2000, three pneumococcal conjugate vaccines including 7-, 10-, and 13-valent conjugate vaccines (PCV7, PCV10, and PCV13), which target some serotypes of pneumococci, have been successively introduced for preventing invasive pneumococcal disease in numerous countries [4–6]. As a result, there has been a remarkable decrease in the incidence of pneumococcal diseases including invasive and noninvasive infections [6–7].

Although PCV7 was firstly introduced into the market in 2008 in mainland China, it has still not been taken into the standard childhood immunization program. People who want to be vaccinated with PCV7 must do so at their own expense and PCV7 is very rarely administered to children. At the time this study was conducted, PCV10 and PCV13 had not yet been introduced into the market in mainland China, thus people there were unable to be vaccinated with these two vaccines. Hence, systematic surveillance of the serotype distribution of *S*,*pneumoniae* is required to assess the necessity of these vaccines.

β-lactam antibiotics are recommended as the primary treatment for pneumococcal infections. The first strain of penicillin-nonsusceptible *S*.*pneumoniae* (PNSSP) was originally detected in 1967 in Australia [8], and subsequently the β-lactam antibiotic-resistant rates of *S*.*pneumoniae* have continued to increase and have become a worldwide concern. Recent reports indicated that the rates of PNSSP in the United States in 2011 and in Asian countries in 2012 were 14.8% and 4.8%, respectively [9–10]. Similarly, the emergence of multidrug-resistant (MDR) *S*.*pneumoniae* isolates has also made the treatment of pneumococcal illnesses even more difficult.

Long-term regional surveillance of pneumococcal isolates is beneficial for developing the right measures to prevent and control pneumococcal infections. Although several reports have monitored the serotype distribution and antimicrobial resistance of *S*.*pneumoniae* in various locations of China, there are few data regarding the overall prevalence of *S*.*pneumoniae* in Shanghai. Therefore, this study aimed to conduct a multicenter surveillance of the serotype distribution, antimicrobial susceptibility, and molecular epidemiology of *S*.*pneumoniae* isolates isolated from children in Shanghai, China.

## Materials and Methods

### Participating centers and clinical isolates

This study was conducted from January to December 2013 in four hospitals: Shanghai Children’s Hospital, Shanghai Children’s Medical Center, Shanghai Ruijin Hospital, and Shanghai Sixth Hospital. All pneumococcal isolates collected in the four hospitals in 2013 were included in this study, and a total of 284 isolates were collected from children aged 0 to 14 years. No children enrolled in this study had been vaccinated with PCVs prior to or during the study. Only one isolate per patient was included. Of the isolates, 270 were isolated from sputum, 5 from bronchoalveolar lavage fluid, 4 from blood, 3 from cerebral spinal fluid (CSF), and 2 from ear secretions. This study was approved by the Ethics Committee of Shanghai Children’s Hospital. Written informed consent was obtained from the patients’ guardians on behalf of the children enrolled in this study.

### Microbiological methods

Sputum samples were collected by using suction and other samples were collected by conventional methods which were recommended in the clinic. All samples were transported to the department of clinical microbiology within 2h and inoculated onto agar plates supplemented with 5% sheep blood. These plates were incubated at 37°C in 5% CO_2_ atmosphere for 18–24 h. Suspected colonies were identified on the basis of typical colony morphology, the presence of alpha hemolysis and Gram positive diplococci. Presumptive isolates were finally confirmed by the optochin sensitivity test (Oxoid, Basingstok, UK) and the bile solubility test [11]. All isolates were stored at -80°C in 40% sterilized glycerin bouillon for further analysis.

### Serotyping

Pneumococcal isolates were serotyped by multiplex polymerase chain reaction (MP-PCR) as described previously [12]. If isolates could not be typed by MP-PCR, they were further analyzed by capsule-quellung reaction [13] with a set of antisera from the Statens Serum Institute (Copenhagen, Denmark). Serotypes that could not be identified by MP-PCR and capsule-quellung reaction were classified as non-typeable. The coverage rates of the PCV7 (serotype 4, 6B, 9V, 14, 18C, 19F, and 23F), PCV10 (PCV7 plus serotypes 1, 5, and 7F), and PCV13 (PCV10 plus serotypes 3, 6A, and 19A) vaccines were also estimated.

### Antimicrobial susceptibility test

Susceptibility test of all isolates against penicillin (PEN), cefuroxime (CXM), ceftriaxone (CRO), erythromycin (ERY), azithromycin (AZM), clindamycin (CLI), levofloxacin (LEV), moxifloxacin (MXF), vancomycin (VAN) and trimethoprim-sulfamethoxazole (SXT) were conducted by determining the minimum inhibitory concentrations (MICs) using the broth microdilution method. The breakpoints used for interpretation were recommended by the Clinical and Laboratory Standards Institute (CLSI) 2014 [14]. *S*.*pneumoni*ae ATCC 49619 was used as the control strain. Isolates that were resistant to three or more classes of antimicrobial agents were defined as MDR *S*.*pneumoniae*.

### Multilocus sequence typing (MLST)

To determine the relationships between sequence types (STs) and serotypes, a total of 267 isolates with certain serotypes were investigated by MLST analysis. Briefly, the pneumococcal MLST scheme used the internal fragments of seven housekeeping genes (*aroE*, *gdh*, *gki*, *recP*, *spi*, *xpt*, and *ddl*), which were amplified by PCR as previously described [15]. The STs were obtained by sequencing and submitting the sequences to the *S*.*pneumoniae* MLST database (http://pubmlst.org/spneumoniae/) for identification. Then the STs were compared with Pneumococcal Molecular Epidemiology Network (PMEN) clones (http://www.pneumogen.net/pmen/).

### Statistical analysis

The antimicrobial susceptibility data were analyzed using WHONET 5.6 software. The chi-square test or Fisher’s exact test o in the Statistical Package for Social Science (SPSS) for Windows, Version 16.0 (SPSS Inc., Chicago, IL, USA) were performed to test the significance of the data. A two-tailed cutoff of *P*<0.05 was considered to be statistically significant.

## Results

### Serotype distribution

Among the 284 pneumococcal isolates, 267 isolates (94.0%) were properly serotyped. The remaining 17 isolates were classified as non-typeable. The most common serotypes were 19F (33.5%), 19A (14.1%), 23F (12.0%), 6A (8.8%), 15B/C (7.7%), 6B (6.7%), and 14 (6.0%), which accounted for 88.8% of the isolates. The coverage rates of PCV7, PCV10, and PCV13 were 58.6%, 59.4%, and 85.1%, respectively.

### Antimicrobial susceptibility

The rates of susceptible, intermediate, and resistance of *S*.*pneumoniae* isolates are listed in Table 1. The prevalent rates of penicillin-resistant *S*.*pneumoniae* (PRSP) were 10.7% and 66.7% in non-meningitis and meningitis isolates, respectively. The proportions of ceftriaxone resistance were 28.1% in non-meningitis isolates and 33.3% in meningitis isolates. All isolates showed high resistant rates to cefuroxime, erythromycin, azithromycin, clindamycin, and trimethoprim-sulfamethoxazole. However, most isolates were susceptible to levofloxacin, moxifloxacin, and vancomycin.

**Table 1:** Antimicrobial susceptibility of the 284 pneumococcal isolates to 10 common antimicrobial agents.

Antimicrobial agents^1^	Isolates	Susceptibility (%)			MIC(μg/mL)		
		S	I	R	MIC50	MIC90	Range of MIC
PEN							
Meningitis	3	33.3	0	66.7	0.5	2	0.06–2
Non-meningitis	281	80.0	9.3	10.7	1	8	<0.06–16
CXM	284	12.7	6.3	81.0	8	>32	<0.06 to >32
CRO							
Meningitis	3	66.7	0	33.3	0.25	4	0.25–4
Non-meningitis	281	55.5	16.4	28.1	1	16	<0.06–32
ERY	284	2.8	0	97.2	>32	>32	<0.06 to >32
AZM	284	2.8	0.4	96.8	>32	>32	<0.06 to >32
CLI	284	3.5	0	96.5	>32	>32	<0.06 to >32
LEV	284	99.3	0.4	0.4	0.25	1	<0.06–8
MXF	284	100	0	0	0.125	0.5	<0.06–1
VAN	284	100	-	-	<0.06	0.25	<0.06–0.5
SXT	284	12.7	7.4	79.9	8/152	16/304	0.06/1.2-16/304

^**1**^ PEN: penicillin, CXM: cefuroxime, CRO: ceftriaxone, ERY: erythromycin, AZM: azithromycin, CLI: clindamycin, LEV: levofloxacin, MXF: moxifloxacin, VAN: vancomycin, SXT: trimethoprim-sulfamethoxazole

As depicted in Table 2, serotypes 14, 15B/C, and 19F showed higher resistant rate to penicillin. The highest resistant rate of ceftriaxone was observed in serotype 19F. Furthermore, all serotypes exhibited high resistant rates to ceftriaxone, macrolides and trimethoprim-sulfamethoxazole. The isolates covered by PCV7 showed a significantly higher rate of cefuroxime resistance than those not covered by this vaccine (90.6% vs 68.9%, respectively; *χ* ^2^ = 21.098, *P*<0.01). A similar phenomenon was observed for ceftriaxone (43.0% vs 11.1%, respectively; *χ* ^2^ = 35.765, *P*<0.01), but there was no significant difference between the PCV7 and non-PCV7 groups regarding penicillin resistance (12.7% vs 9.6%, respectively; χ^2^ = 0.691, *P* = 0.406).

**Table 2:** Antimicrobial resistance of *S*.*pneumoniae* against antimicrobial agents among different serotypes.

	Number of isolates	PEN^1^	CXM^1^	CRO^1^	ERY^1^.	AZM^1^	CLI^1^.	LEV^1^	SXT^1^
		No. (%)	No. (%)	No. (%)	No (%)	No. (%)	No (%)	No. (%)	No. (%)
19F	95	17 (17.9)	91 (95.8)	57 (60)	93 (97.9)	93 (97.9)	92 (96.8)	0 (0)	92 (96.8)
19A	40	3 (7.5)	39 (97.5)	6 (15.0)	40 (100)	40 (100)	40 (100)	1 (2.5)	39 (97.5)
23F	34	0 (0)	30 (88.2)	2 (5.9)	34 (100)	34 (100)	34 (100)	0(0)	32 (94.1)
6A	25	0 (0)	17 (68.0)	0 (0)	25(100)	25(100)	25(100)	0 (0)	14 (56.0)
15B/C	22	4 (18.2)	18 (81.8)	4 (18.2)	20 (90.9)	19 (86.4)	20 (90.9)	0 (0)	16 (72.7)
6B	19	2 (10.5)	15 (78.9)	5 (26.3)	19 (100)	19 (100)	19 (100)	0 (0)	11 (57.9)
14	17	3 (17.6)	9 (52.9)	2 (11.8)	17 (100)	17 (100)	17 (100)	0 (0)	5 (29.4)
Others	15	1 (6.7)	2 (13.3)	1 (6.7)	11 (73.3)	11 (73.3)	10 (66.7)	0 (0)	6 (40.0)
NT^2^	17	2 (11.8)	9 (52.9)	3 (17.6)	17 (100)	17 (100)	17 (100)	0 (0)	12 (70.6)
Total	284	32 (11.3)	230(81.0)	80 (28.2)	276(97.2)	275(96.8)	274(96.5)	1(0.4)	227(79.9)

^**1**^ PEN: penicillin, CXM: cefuroxime, CRO: ceftriaxone, ERY: erythromycin, AZM: azithromycin, CLI: clindamycin

LEV: levofloxacin, SXT: trimethoprim-sulfamethoxazole

^2^ NT: non-typeable

The resistance patterns of the pneumococcal isolates from the current study are shown in Table 3. About 88.0% (250/284) of the isolates were defined as MDR and the resistance pattern of CXM-ERY-AZM-CLI-SXT was commonly identified (49.6%, 124/250). Of the 250 MDR isolates, 19F (n = 93, 37.2%), 19A (n = 39, 15.6%), 23F (n = 33, 13.2%), and 6A (n = 23, 9.2%) were the most common serotypes. The most common resistance patterns of serotypes 19F and 19A were CXM-CRO-ERY-AZM-CLI-SXT and CXM-ERY-AZM-CLI-SXT, respectively. PCV13 covered 86.4% (216/250) of the MDR isolates, which was higher than that for PCV7 (60.4%, 151/250).

**Table 3:** Antimicrobial resistance pattern of 284 pneumococcal isolates.

Resistance patterns^1^	NO.	Proportion (%)	Related serotypes (no.)
—	6	2.1	15B/C(2), 19F(1), 3(1), 1(1), 18(1)
SXT	2	0.7	19F(1), 3(1)
ERY-AZM	1	0.4	3(1)
ERY-AZM-CLI	25	8.8	14(8), 6B(4), 6A(2), 15B/C(2), 3(2), 19A(1), 23F(1), 7F(1), 33F(1), untyped(3)
ERY-AZM-CLI-SXT	20	7.0	6A(6), 23F(3), 19F(2), 3(2), 9V(1), 34(1), untyped(5)
CXM-ERY-AZM-CLI	23	8.1	6A(9), 6B(4), 14(3), 19F(2), 23F(1), 15B/C(1), 15A(1), untyped(2)
CXM-ERY-CLI-SXT	1	0.4	15B/C(1)
PEN-CXM-ERY-AZM-CLI	1	0.4	14(1)
CXM-ERY-AZM-CLI-SXT	124	43.7	19F(32), 19A(32), 23F(27), 15B/C(12), 6A(8), 6B(6), 14(3), untyped(4)
CXM-CRO-ERY-AZM-SXT	1	0.4	19F(1)
PEN-CXM-CRO-ERY-AZM-CLI	1	0.4	15B/C(1)
CXM-CRO-ERY-AZM-CLI-SXT	48	16.9	19F(39), 19A(3), 6B(3), 23F(2), untyped(1)
CXM-ERY-AZM-CLI-LEV-SXT	1	0.4	19A (1)
PEN-CXM-CRO-ERY-AZM-CLI-SXT	30	10.6	19F(17), 19A(3), 15B/C(3), 6B(2), 14(2), 3(1), untyped(2)

^1^ PEN: penicillin, CXM: cefuroxime, CRO: ceftriaxone, ERY: erythromycin, AZM: azithromycin, CLI: clindamycin, LEV: levofloxacin, SXT: trimethoprim-sulfamethoxazole

### MLST

Among the 267 serotyped isolates, 65 STs were identified by MLST analysis. The four predominant STs were ST271 (24.7%, 66/267), ST320 (11.2, 30/267), ST81 (9.7%, 26/267), and ST3173 (5.2%, 14/267). Most of the ST271 isolates were serotyped as 19F, and all ST320, ST81 and ST3173 isolates were related to serotypes 19A, 23F and 6A, respectively. Finally, all the isolates with ST3397 were serotyped as 15B/C, and all the isolates with ST90 were serotyped as 6B (Table 4).

**Table 4:** Sequence types of 267 pneumococcal isolates among different serotypes.

Serotypes	No.	Sequence types (no.)
19F	95	271(65), 236(10), 2648(6), 1464(4), 283(2), 2116(2), 8227(2), 876(1), 983(1), 9822(1), 9877(1)
19A	40	320(30), 9630(2), 276(1),416(1), 3111(1), 7964(1), 9878(1), 9879(1), 9880(1), 9881(1)
23F	34	81(26), 242(3), 271(1), 342(1), 802(1), 880(1), 6942(1)
6A	25	3173(14), 8916(3), 982(2), 855(1), 2912(1), 6918(1), 9819(1), 9820(1), 9821(1)
15B/C	22	3397(10), 83(5), 199(2), 2760(1), 6011(1), 8905(1), 9882(1), 9883(1)
6B	19	90(11), 902(2), 386(1), 2757(1), 4757(1), 8616(1), 9776(1), 9777(1)
14	17	876(9), 200(4), 143(2), 7964(1), 875(1)
Others	15	6875(3), 180(2), 166(1), 191(1), 505(1), 673(1), 1902(1), 2296(1), 3058(1), 7964(1), 8173(1), 9778(1)
Total	267	

A comparison of the isolates with the PMEN clones (at least six of seven MLST alleles in common) revealed that 47.2% (126/267) of the isolates were assigned to international clones or their single locus variants (SLVs).The four international antibiotic-resistant clones were Taiwan^19F^-14, Spain^23F^-1, Spain^6B^-2, and Taiwan^23F^-15. Furthermore, the Taiwan^19F^-14 clone comprised 5 STs, including ST236 (n = 10), ST271 (n = 66), ST283 (n = 2), ST2116 (n = 2), and ST8227 (n = 2). Spain^23F^-1clones were also frequently identified in this study and this group of isolates included 2 STs, the original Spain^23F^-1 clone ST81 (n = 26) and SLV ST83 (n = 5). Isolates related to antibiotic-resistant PMEN clones accounted for 55.2% of PNSSP, including 53.1% of PRSP and 57.7% of penicillin-intermediate *S*.*pneumoniae* (PISP).

## Discussion

*S*.*pneumoniae* is one of the important pathogens causing pediatric infections, especially in low-income countries. The serotype distribution and rates of antimicrobial resistance vary across different affected populations and change over time [16]. This multicenter surveillance study demonstrated that the most common serotypes in the pre-vaccine area covered in this study were 19F, 19A, 23F, 6A, 15B/C, 6B, and 14 and the coverage rate of PCV7 was 58.6%, which was similar to other reports about invasive and non-invasive pneumococcal infections in 2010 [17–18]. Thus it can be seen that serotype19F which is included in PCV7 is still responsible for the highest number of pneumococcal infections in mainland China, even though non-vaccine serotypes make up a large proportion of infection isolates. The prevalence of serotype 19F isolates might be the result from the fact that PCV7 has not been widely used and children are vaccinated with it on a voluntary basis.

In the present study, the rate of PNSSP in non-meningitis isolates in 2013 in Shanghai was 20.0%, which was still higher than that reported in Beijing (0.7%) in 2010 [19] and in Asia (4.6%) from 2008 to 2009 [10] using the same CLSI criteria. Furthermore, the ceftriaxone non-susceptible rate was higher than that of penicillin and the resistance rates of some serotypes (especially for 19F) to ceftriaxone were higher than those to penicillin as well. This phenomenon may be the result of the widespread use of third-generation cephalosporins. A cross-sectional study conducted in 2013 showed that the antibiotics commonly used by decreasing frequency were macrolides, third generation cephalosporins, second-generation cephalosporins, first generation cephalosporins, and β-lactams in Chinese hospitals [20]. In fact, the situation of inappropriate use and abuse of cephalosporins has been reported in China [21–22]. Macrolides are another choice for the treatment of pneumococcal infections, but current data showed that resistance to erythromycin was very high in Asia in 2012 (72.7%) and the highest rates were observed in China (96.4%), Taiwan (84.9%), and Vietnam (80.7%) [10]. A similar result (>95%) was also detected in this study, which suggested that macrolides are not suitable for the clinical treatment of pneumococcal disease in China. As mentioned above, in mainland China, macrolides are frequently prescribed to children with infectious diseases. This practice has not been curbed despite a growing awareness of antimicrobial resistance, which may provide an explanation for the reason why the high resistant rates of macrolides remain high among isolates from children [23].

MDR, which threatens the treatment of pneumococcal diseases, is of considerable concern. In an Asian Network for Surveillance of Resistant Pathogens (ANSORP) study of clinical isolates obtained from 2008 to 2009, the overall MDR rate was 59.3%, with the highest MDR rate being 83.3% in China, followed by 75.5% in Vietnam [10]. In 2010, a previous study of invasive pneumococcal isolates in China demonstrated that 89.5% of the isolates were MDR [18]. In our study, 88.0% of the isolates were defined as MDR, which was similar to the reports above. Here, PCV7, PCV10, and PCV13 covered 60.4%, 60.4%, and 86.4% of the MDR isolates, respectively, which illustrated that PCV13 could be useful for controlling the spread of MDR pneumococcal isolates.

A molecular analysis of these pneumococcal isolates showed that ST271, ST320, and ST81 were commonly isolated in this study. This finding was similar to other reports about invasive and non-invasive isolates in other regions of China [24–25]. In this study, four international antibiotic-resistant clones responsible for the spread of antimicrobial resistance were identified and they accounted for 55.2% of PNSSP. Of the four identical clones, the Taiwan^19F^-14 (ST236) clone, an international MDR *S*.*pneumoniae* clone, was the predominant clone among all serotyped isolates. As was reported, the Taiwan^19F^-14 clone was firstly isolated from a Taiwanese hospital in 1993 [26] and then spread internationally [27]. Since the late 1990s in China, the Taiwan^19F^-14 clone has become prevalent prior to the introduction of PCV7 and clonal spread has played an important role in the emergence of antimicrobial resistance in Shanghai [28]. However, long-term monitoring data suggested that ST271 and ST320 replaced ST236, an original Taiwan^19F^-14 clone, as the cause of disease in children and contributed to antimicrobial resistance [25, 29–30]. Further analysis of this study demonstrated that the Taiwan^19F^-14 clone had a 34.1% penicillin non-susceptible rate, which was lower than the 60.8% rate in Suzhou, China [24]. ST271 was a SLV of ST236, and constituted the majority of STs included in the Taiwan^19F^-14 clone. This may be associated with the low nonsusceptibility rate to penicillin, but Taiwan^19F^-14 clone still comprised 48.3% of PNSSP isolates, which was mainly responsible for penicillin -resistance. Therefore, given this phenomenon, the need for continued molecular surveillance should be emphasized.

In conclusion, the current data demonstrated that serotype 19F, 19A, 23F, and 6A were commonly isolated from children and that these isolates were highly resistant to β-lactams and macrolides. Molecular epidemiological surveillance showed that the Taiwan^19F^-14 clone played a predominant role in the dissemination of pneumococcal isolates in Shanghai, China. Therefore, long-term regional surveillance is essential for providing optimal antimicrobial therapy, monitoring molecular clones, and formulating an adequate vaccination strategy for *S*.*pneumoniae* infections.
